# NRF2-mediated persistent adaptation of oesophageal adenocarcinoma cells to HER2 inhibition

**DOI:** 10.1038/s41388-025-03459-0

**Published:** 2025-06-05

**Authors:** Wei Zhang, Jiaqing Lang, Sorayut Chattrakarn, Chun Wai Wong, Shiyang Li, Karmern Kan, Hongcai Liu, Wenchao Gu, Jingwei Zhang, Jukka Westermarck, Alan J. Whitmarsh, Andrew D. Sharrocks, Cathy Tournier

**Affiliations:** 1 Division of Cancer Sciences, School of Medical Sciences, Faculty of Biology, Medicine and Health (FBMH), University of Manchester, Manchester, UK; 2 Turku Bioscience Centre, University of Turku and Åbo Akademi University, Turku, Finland; 3 Division of Molecular and Cellular Function, School of Biological Sciences, FBMH, University of Manchester, Manchester, UK; 4 Lydia Becker Institute of Immunology and Inflammation, FBMH, University of Manchester, Manchester, UK; 5 Massachusetts General Hospital Cancer Center, Harvard Medical School, MA Boston, USA; 6 Division of Cell Matrix Biology and Regenerative Medicine, School of Biological Sciences, FBMH, University of Manchester, Manchester, UK; 7 Department of Neurology, Sankt Rochus Kliniken Bad Schönborn, Bad Schönborn, Germany; 8 Department of Artificial Intelligence Medicine, Chiba University, Chiba, Japan; 9 Whitehead Institute for Biomedical Research, MA Cambridge, USA

**Keywords:** Targeted therapies, Transcription, Mechanisms of disease, Oesophageal cancer

## Abstract

The human epidermal growth factor receptor 2 (HER2, also known as ERBB2) is a commonly over-expressed oncoprotein in oesophageal adenocarcinoma (OAC). Nonetheless, HER2-blocking agents have failed to significantly improve the outcome for OAC patients, despite achieving striking clinical success in breast cancer. To address this conundrum, we investigated how resistance progressively emerges when HER2 is targeted. We discovered that OAC cell lines that are capable of surviving in the presence of the dual HER1/HER2 tyrosine kinase inhibitor lapatinib exhibit a significant increase in the protein level of nuclear factor erythroid 2-related factor 2 (NRF2). Indeed, NRF2 knockdown enhanced the cytotoxic effect of lapatinib, while increased NRF2 expression in OAC cells reduced their sensitivity to HER inhibition. Furthermore, prolonged overexpression of NRF2 made OAC cell lines increasingly dependent on NRF2 for growth. Further analyses indicated that the activation of NRF2-mediated transcription that was associated with lapatinib-induced persistent and resistant phenotypes coincided with a subsequent increase in glutathione metabolism. Importantly, lapatinib resistant OAC xenografts become exquisitely sensitive to pharmacological inhibition of the NRF2 pathway. Together, these findings highlight a promising therapeutic strategy for treating refractory OAC by targeting the NRF2 pathway in combination with receptor tyrosine kinase inhibition.

## Introduction

Oesophageal adenocarcinoma (OAC) is the most common histological subtype of oesophageal cancer and a major cause of cancer-related mortality in Europe and North America [1]. Around a quarter of patients exhibit high expression of the human epidermal growth factor receptor 2 (HER2, also known as ERBB2) as a result of gene amplification or mutation [2, 3]. HER2 drives oncogenic signalling through forming homo- and heterodimers with other epidermal growth factor (EGF) family receptors such as HER1/EGFR and HER3/ERBB3 [4]. Hence, HER2 constitutes an actionable therapeutic target for treating HER2-positive diseases. Notably, the monoclonal antibody trastuzumab and small molecule inhibitors of HER1/HER2/HER3 tyrosine kinases, such as lapatinib and neratinib, have transformed the care for patients with HER2-overexpressing breast cancer [5]. In contrast, clinical trials of HER2-directed therapies have achieved limited benefit for OAC patients [6–9]. These disappointing observations raised the question of why HER2 blocking agents failed to improve the survival of OAC patients, despite achieving clinical success in breast cancer. This may, in part, be due to the aggressive nature of oesophageal cancer and the difficulty in detecting early stage tumours [1]. However, it may also result from the existence and persistence of a subset of cancer cells that can survive initial receptor tyrosine kinase (RTK) inhibitor therapies and from which drug-resistant mutant cells eventually emerge to give rise to metastatic relapses [10–13].

It has become increasingly clear that cancer persistence induced by drug treatment initially occurs through epigenetic modifications of slow-proliferating cells rather than selection for resistant mutations [14–16]. In particular, we previously demonstrated that OAC cells continuously exposed to lapatinib underwent a gradual reprogramming of their transcriptome through widespread remodelling of the accessible chromatin landscape [17]. Accordingly, the survival advantage acquired by those OAC cells that are in a drug-tolerant persistent (DTP) state reverted to a drug-sensitive phenotype upon interruption of the treatment [17]. Alongside their high level of epigenetic plasticity, persistent cancer cells exhibit a distinct intrinsic ability to re-enter the cell cycle and resume proliferation, even under continuous drug challenge [12, 14, 17, 18]. This differs from the cancer dormancy status, which refers to cancer cells that stay quiescent for extended periods of time, regardless of the use of pharmaceutical agents.

Additionally, persistent cancer cells undergo significant metabolic changes to survive under therapeutic stress conditions [19]. For example, in our previous study, we found that transcriptional adaptation to lapatinib treatment through transient widespread chromatin remodelling shifted the OAC cells towards oxidative phosphorylation [17]. This phenotype has been observed across a variety of drug-induced persistent cancer cell lines [20]. In this context, it is generally recognised that cancer cells transitioning into the DTP state develop unique vulnerabilities as a consequence of metabolic reprogramming [19]. Notably, persistent cells are highly dependent on glutathione peroxidase 4 (GPX4) which uses glutathione (GSH) as a cofactor to prevent iron-dependent lipid peroxides causing irreparable damage to the plasma membrane and ferroptotic death [21, 22]. Similarly, cancer cells that developed tolerance to paclitaxel were found to be sensitive to ferroptosis induced by inhibitors of system xc^−^ cystine/glutamate antiporter (xCT) which decrease intracellular GSH levels [23]. Collectively, these observations have offered a novel perspective of treatment for eliminating persistent cancer cells and preventing disease recurrence in cancer patients [24].

To further understand the mechanism underlying the acquisition of drug resistance through persistence, we sought to decipher the transcriptional adaptation of OAC cells to the anti-tumour activity of HER2-targeting agents in order to identify novel actionable therapeutic opportunities.

## Results

### Acquisition of lapatinib tolerance is associated with increased NRF2-mediated transcriptional regulation

**Fig. 1:**
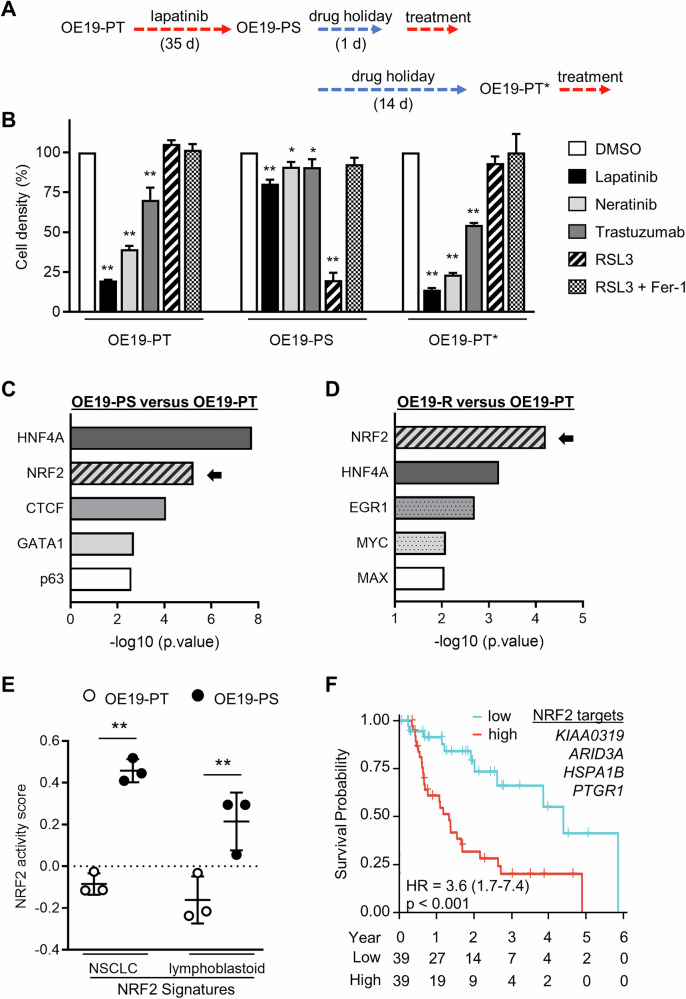
Lapatinib-tolerant OE19 persister cells exhibit NRF2-mediated transcriptional activation. **A** OE19-PT cells were treated with lapatinib (500 nM) for 35 days and released from therapy for 1 day (OE19-PS) or 14 days (OE19-PT*) prior to being re-exposed to lapatinib (500 nM), neratinib (10 nM) or trastuzumab (10 µg/mL) for 3 days, or incubated with the GPX4 inhibitor RSL3 (500 nM) with or without the ferroptosis inhibitor Ferrostatin-1 (Fer-1, 1 µM) for 1 day. **B** Cell density was measured by crystal violet staining. One-way ANOVA was utilised to analyse statistical differences between treated versus mock treated cells with DMSO (*n* = 3). **C**, **D** X2K webtool was utilised to predict transcription factors interacting with regulatory binding regions of DEGs upregulated in OE19-PS (panel **C**) or in lapatinib-resistant OE19-R (panel **D**) cells compared with the OE19-PT cell line. **E** RNA-sequencing datasets were utilised to assess the level of NRF2 activity in OE19-PT and OE19-PS cells by GSVA of NRF2 core target gene signatures derived from human NSCLC and lymphoblastoid cells. Unpaired t-test was utilised to analyse statistical differences (*n* = 3). **F** Kaplan–Meier plot survival curves comparing subjects in the TCGA OAC cohort based on risk scores established from four NRF2 target genes. Samples were divided into two groups, high (red) and low (blue), based on the median value of risk scores. Cox regression was used to calculate the hazard ratio (HR) and the *p*-value.

To investigate how resistance of OAC to HER2-targeted therapy emerges from DTP states, we studied the OE19 cancer cell line that closely resembles HER2-amplified OAC patient derived samples [17]. Previously, we showed that a population of OE19-parental (OE19-PT) cells could enter a DTP state to evade the strong selective pressure of a high concentration (500 nM) of lapatinib for 35 days, at which point the cells we able to resume proliferation demonstrating that they were not permanently growth-arrested [17]. In this study, we employed the same protocol to generate OE19-persistent (OE19-PS) cells, which remained tolerant to lapatinib upon re-exposure to the drug for 3 days after being released from the initial therapy for 1 day (Fig. 1A, B). Likewise, OE19-PS cells displayed decreased sensitivity towards the structurally distinct pan-HER kinase inhibitor neratinib (10 nM), which is an irreversible inhibitor of HER1, HER2 and HER4, and the antagonist monoclonal HER2 antibody trastuzumab (10 μg/ml) (Fig. 1B). Conversely, OE19-PS cells were exquisitely sensitive to GPX4 inhibition by RSL3, consistent with the specific vulnerability of persistent cancer cells to ferroptotic inducers (Fig. 1B) [24]. The protective effect of Ferrostatin-1 (Fer-1) against RSL3 cytotoxicity confirmed that OE19-PS cells incubated with RSL3 were dying through ferroptosis. Consistent with the reversibility of persister states observed in other cancer cell models, the removal of lapatinib for two weeks allowed OE19-PS cells to re-acquire sensitivity to HER2 blockade (Fig. 1A, B) [25]. These cells were named OE19-PT* cells and, similar to the OE19-PT cells, they were insensitive to RSL3-induced ferroptotic death (Fig. 1B).

To elucidate the molecular changes involved in the progressive acquisition of the persister phenotype generated by lapatinib, we analysed the transcriptome of OE19 cells by bulk RNA-sequencing (RNA-seq) after incubation with the drug for 1, 7 and 35 days [17]. Differentially expressed genes (DEGs) in treated versus untreated (day 0) cells were segregated into nine clusters based on similar temporal gene expression patterns (Supplementary Fig. 1A). In order to identify drivers of lapatinib tolerance, we focused on clusters 2 and 9 which comprised transcripts exhibiting sustained increased expression throughout the time course of the experiment. The X2K tool was utilised to predict key transcriptional regulators based on binding interactions with regulatory regions of the corresponding genes [26]. NRF2 and HNF4A were found to be the two most common transcription factors (TFs) predicted to be involved in controlling upregulated DEG sets at day 35 (Fig. 1C). A similar observation was made from analysing publicly available RNA-sequencing datasets obtained from lapatinib-resistant OE19 cells (Fig. 1D) [27]. HNF4A had previously been identified by our group to be required for the acquisition of resistance to HER2 inhibition [17]. Therefore, we focused our attention on NRF2 given its pivotal role in metabolic reprogramming and ferroptotic death [28].

Initially, we validated the increased expression of canonical NRF2 target genes in OE19 cells stimulated with the classical NRF2 activator tertiary butylhydroquinone (tBHQ) (Supplementary Fig. 1B). Then, to get a broader picture, lapatinib-induced global transcriptional changes at day 35 relative to day 1 were compared to a curated database of 3048 RNA-seq datasets from 1458 gene knockdown experiments in human cell lines [29]. Two of the top 5 genetic perturbations that had the most similar transcript signature to that associated with prolonged lapatinib treatment resulted from NRF2 knockdown in the human NRF2-addicted non-small cell lung carcinoma (NSCLC) cell lines H2122 and H460 (Supplementary Fig. 1C). In parallel, we performed gene set variation analysis (GSVA) to compare the level of NRF2 activity in our bulk RNA-sequencing datasets to curated NRF2 gene target signatures consisting of 108 genes from human NSCLC and 207 genes from lymphoblastoid cells (Supplementary Table 1) [30, 31]. We found that NRF2 signatures were consistently upregulated in OE19 persister cells (Fig. 1E). Notably, sixty seven NRF2 regulated genes were detected in clusters 2 and 9 (Supplementary Fig. 2A and Supplementary Table 1). Heatmap visualisation of the data clearly showed a pattern of increased expression of NRF2 targets over the time course of lapatinib treatment (Supplementary Fig. 2B). Conversely, GSVA analysis of pro-ferroptotic and anti-ferroptotic data sets from the FerrDb database, which curates ferroptotic markers manually from the literature, revealed no significant difference in ferroptotic activity score between the OE19-PT and OE19-PS transcriptomes (Supplementary Fig. 2C). Moreover, OE19-PT and OE19-PS cells exhibited a similar level of expression *GPX4* transcript (Supplementary Fig. 2D). To assess the clinical relevance of NRF2 pathway activation in OAC primary tumours, we employed four NRF2 targets (*KIAA0319*, *ARID3A*, *HSPA1B, PTGR1*) from clusters 2 and 9 to calculate the risk score based on datasets from The Cancer Genome Atlas (TCGA). We observed that high risk score was correlated with worse survival of OAC patients (hazard ratio = 3.6; *p* value < 0.001) (Fig. 1F).

**Fig. 2:**
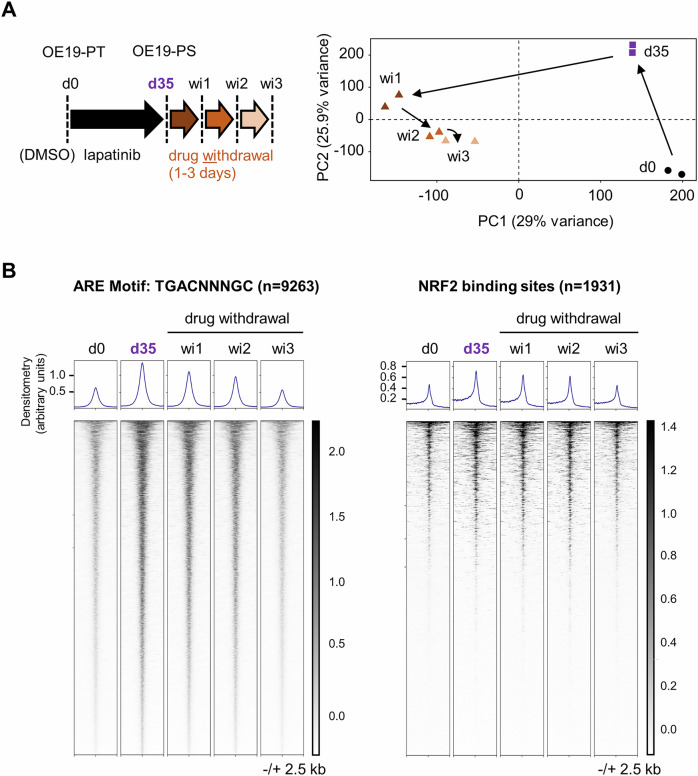
Increased chromatin accessibility of ARE motifs and NRF2-binding sites in OE19 persister cells. **A** Schematic of experimental design. OE19 cells were treated with lapatinib (500 nM) for 35 days and then lapatinib was withdrawn for 1, 2 or 3 days [17]. PCA plot of ATAC-sequencing performed at the indicated time points (*n* = 2). The clustering indicates the reproducibly of samples. **B** Heatmap of differentially accessible ARE-motifs or putative NRF2 binding sites in chromatin after treatment with lapatinib for 35 days and release from therapy relative to mock treated cells with DMSO. NRF2-binding sites were identified from analysing NRF2 ChIP-sequencing datasets from A549 cells [32].

We subsequently utilised genome-wide transposase-accessible chromatin sequencing (ATAC-seq) to profile the open chromatin landscape of mock treated OE19-PT cells and OE19 cells treated with lapatinib for 35 days (OE19-PS) followed by drug withdrawal for 1, 2 or 3 days [17]. Principal component analysis (PCA) of the datasets separated the different groups indicating that they displayed distinct chromatin architectures (Fig. 2A), as previously shown [17]. In particular, we found that regions containing NRF2-binding antioxidant response element (ARE) motifs became more accessible after lapatinib treatment (Fig. 2B). This was confirmed by mapping the accessibility of regulatory binding regions of direct NRF2 target genes that had previously been identified by chromatin immunoprecipitation and sequencing (ChIP–seq) analysis of human A549 lung cancer cells (Fig. 2B) [32]. A549 cells have a high level of NRF2 expression as they harbour a loss-of-function mutation in the *Kelch-like ECH-associated protein 1* (*KEAP1*) gene which encodes a substrate adaptor for a Cullin 3 (CUL3)-based E3 ubiquitin ligase that targets NRF2 for ubiquitination and proteasomal degradation [33]. Increased accessibility of regions containing putative NRF2 binding activity in OE19-PS cells was transient and disappeared 3 days after the cells were released from therapy, confirming the reversibility of the DTP phenotype (Fig. 2B).

Collectively, these results indicated that NRF2 pathway activation was a potentially highly relevant mechanism underpinning the transition of OAC cells from lapatinib sensitivity towards a lapatinib-resistant state.

### Persistent and resistant OE19 cells exhibit vulnerability to NRF2 inhibition

**Fig. 3:**
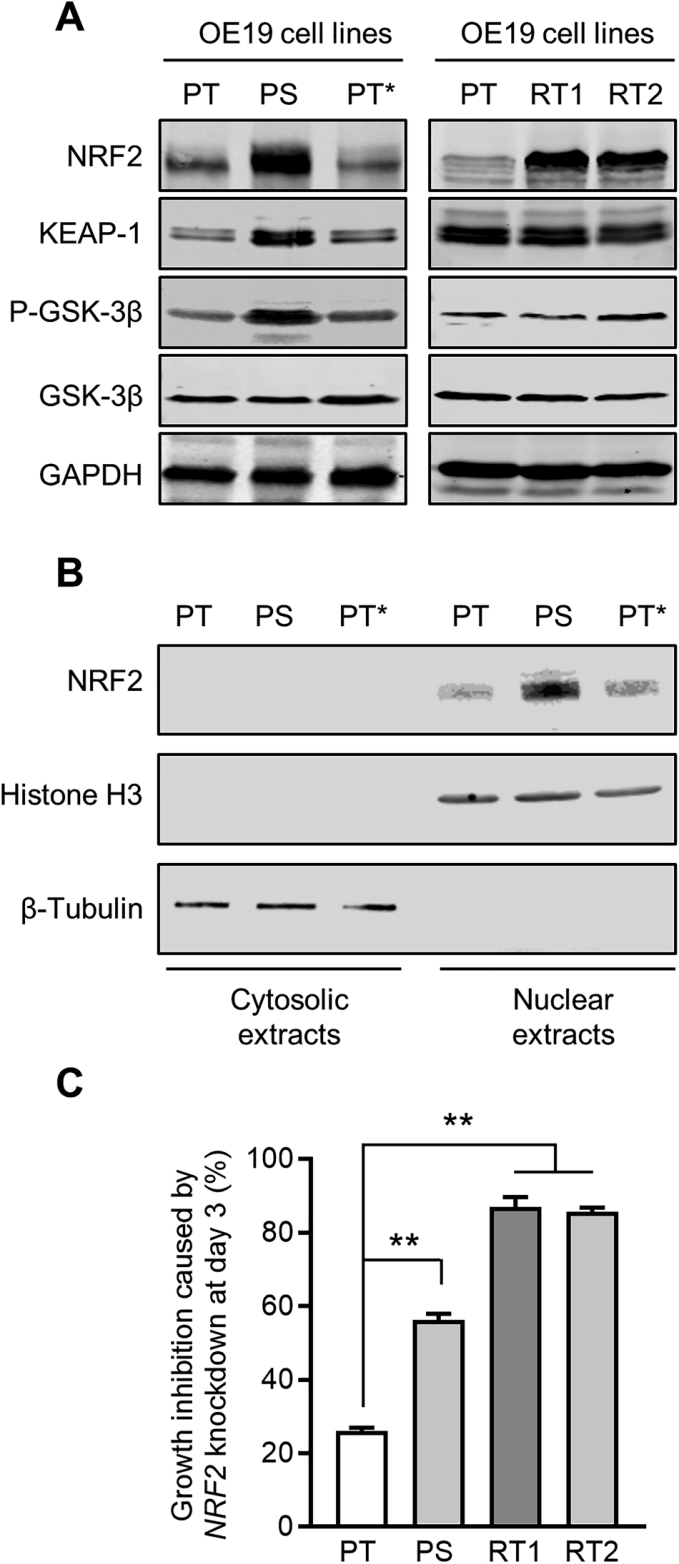
Persistent and resistant OE19 cells exhibit increased vulnerability to NRF2 targeting. **A** Protein lysates of OE19 cell lines were analysed by immunoblot with the indicated antibodies. GAPDH expression was used as loading control. Similar results were obtained in three independent experiments. **B** Immunoblot analysis of NRF2 in nuclear and cytosolic extracts prepared from OE19-PT, OE19-PS and OE19-PS cells with re-acquired lapatinib sensitivity (OE19-PT*). Histone H3 expression was used as a nuclear marker and β-Tubulin as a cytosolic marker indicating no cross-contamination between the nuclear and cytosolic fractions. Similar results were obtained in three independent experiments. **C** Crystal violet staining of OE19 cell lines after NRF2 downregulation by siRNA for 3 days. One-way ANOVA was utilised to analyse statistical differences between growth inhibition caused by NRF2 silencing (siNRF2 treated) in persistent and resistant versus parental cells (*n* = 3).

In addition to generating persistent cells, stable resistance to lapatinib (IC50 value > 1 μM) was achieved by continuously exposing OE19-PT cells to 500 nM lapatinib for 1 year. Like OE19-PS cells, OE19-RT1 (a polyclonal resistant line) and OE19-RT2 (a monoclonal resistant line derived from OE19-RT1) cells were also resistant to neratinib (10 nM) and trastuzumab (10 μg/ml) (Supplementary Fig. 3A), but highly sensitive to ferroptosis by GPX4 inhibition (Supplementary Fig. 3B). We found that the protein level of NRF2 was markedly increased in OE19-PS and OE19-RT cell lines compared with OA19-PT cells (Fig. 3A and Supplementary Fig. 4). We further demonstrated that NRF2 was detected principally in nuclear fractions, consistent with the rapid translocation of NRF2 into the nucleus following synthesis (Fig. 3B). Importantly, OE19-PT* cells, which had re-acquired normal sensitivity to lapatinib treatment, exhibited a relatively lower level of NRF2 similar to the parental cell line (Fig. 3A, B).

Our transcriptomic data analysis indicated that NRF2 protein was not regulated at the transcriptional level. Moreover, increased NRF2 protein expression in lapatinib-persistent and -resistant cell lines was not induced by a reduction in KEAP1 expression (Fig. 3A). In fact, the level of KEAP1 expression was unchanged in the resistant cells and may even be increased in OE19-PS compared with OE19-PT and OE19-PT* cells, although this observation did not reach statistical significance (Supplementary Fig. 4). An alternative mechanism to regulate NRF2 stability is via its phosphorylation by GSK-3β independently of KEAP1 [34]. We therefore investigated whether GSK-3β activation was altered in persistent or resistant OE19 cells. We found that the OE19-PS cells exhibited an elevated level of GSK-3β phosphorylation at Ser9, indicative of low GSK-3β activity [35], while levels in the resistant cells did not change (Fig. 3A and Supplementary Fig. 4). However, inhibition of GSK-3 activity did not increase NRF2 protein levels in OE19-PT cells (Supplementary Fig. 5A), indicating that GSK-3β activity was unlikely to play a role in controlling the level of NRF2 in OAC. We subsequently employed siRNA to deplete NRF2 in OE19 cells (Supplementary Fig. 5B). We found a modest effect on growth in the OE19-PT cells, but a much more significant effect in the persistent cells and an even stronger growth suppressive effect in OE19-RT cells (Fig. 3C). This suggests that OE19 cells become more dependent on NRF2 expression for growth as the period of raised NRF2 levels is extended.

**Fig. 4:**
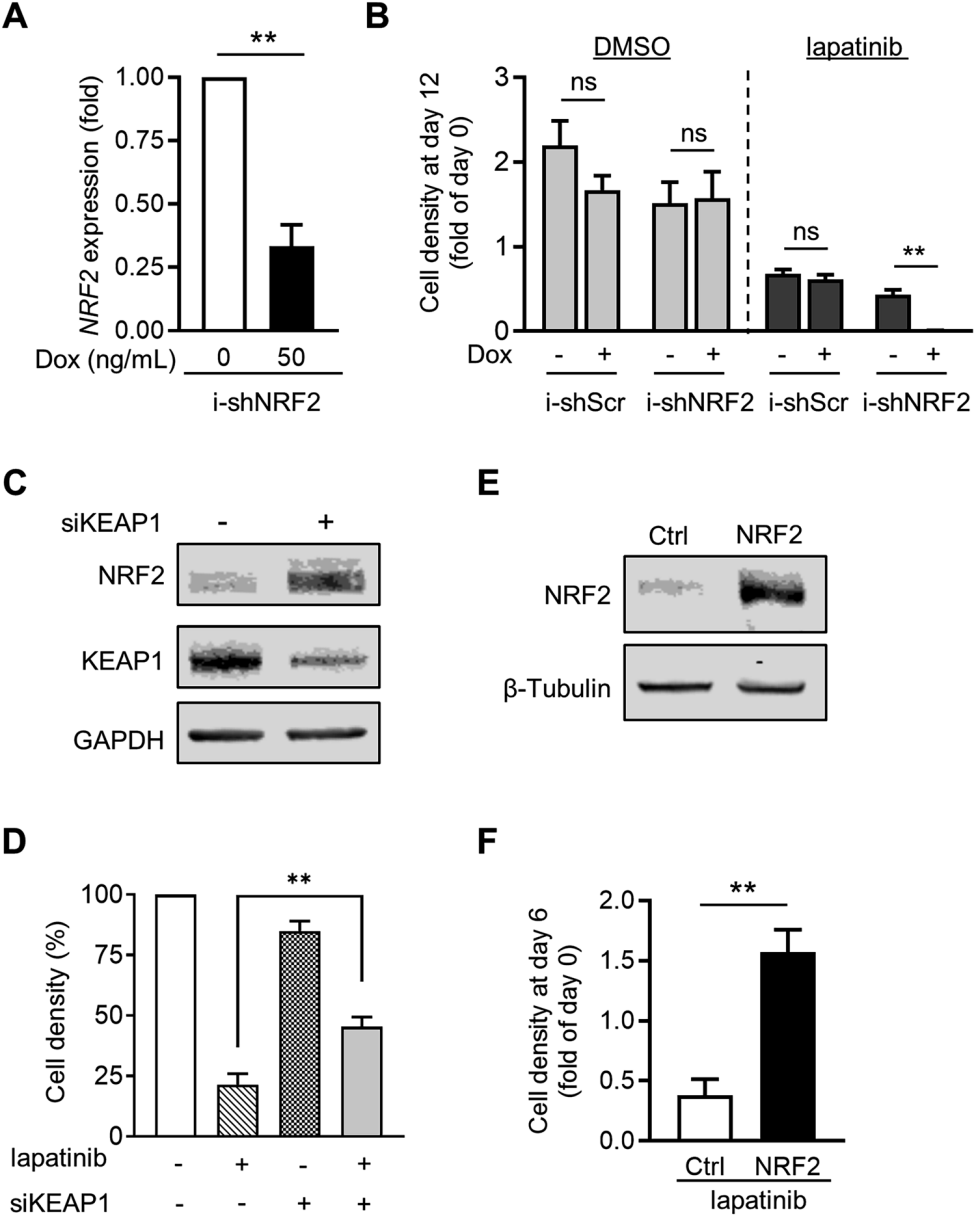
NRF2 overexpression is essential for adaptation of OE19-PT cells to lapatinib. **A** *NRF2* transcript levels were measured by qPCR to demonstrate decreased expression in OE19-PT cells stably transduced with doxycycline inducible shNRF2 (i-shNRF2) encoding lentiviral particles after incubation with 50 ng/mL doxycycline (Dox) for 24 h (*n* = 3). **B** OE19-PT cells expressing inducible shScr or shNRF2 were incubated with DMSO or with lapatinib (500 nM), without or with doxycycline (Dox; 50 ng/mL) for 12 days. Cell density was quantified by crystal violet staining. The data are expressed as fold relative to the day drugs were added (day 0) (*n* = 3). **C** Immunoblot analysis showing increased level of NRF2 expression in OE19-PT cells silenced with siKEAP1 (+) RNA for 24 h. OE19-PT cells transfected with scrambled siScr (−) RNA were used as control. GAPDH expression was used as loading control. Similar results were obtained in two independent experiments. **D** Quantification of cell density by crystal violet staining of OE19-PT cells silenced with siScr (−) or siKEAP1 (+), alone or in combination with lapatinib treatment (500 nM) for 3 days (*n* = 4). **E** Immunoblot analysis showing increased level of NRF2 expression in OE19-PT cells stably transduced with NRF2 encoding lentiviral particles. β-Tubulin expression was used as loading control. Similar results were obtained in three independent experiments. **F** Quantification of cell density by crystal violet staining of Ctrl (empty vector) and NRF2 over-expressing OE19-PT cells treated with lapatinib (500 nM) for 6 days. The data are expressed as fold relative to the day lapatinib was added (day 0) (*n* = 3). Statistical analyses were performed by one-way ANOVA (**D**) or unpaired t-test (**A**, **B**, **F**).

Next, we assessed the requirement of NRF2 for OE19 cells to acquire a persistent phenotype. This was achieved by generating OE19-PT cells carrying a doxycycline inducible shRNA targeting NRF2 (i-shNRF2). We demonstrated that doxycycline reduced *NRF2* transcript levels in OE19-PT cells harbouring i-shNRF2 to a similar extent as we observed for siRNA targeting NRF2 (Fig. 4A and Supplementary Fig. 5B). As expected, doxycycline did not affect the growth of OE19-PT cells transduced with a scrambled shRNA control (i-shScr) (Fig. 4B). Similarly, the downregulation of NRF2 induced by doxycycline did not have a significant effect on the long-term proliferation of OE19-PT cells (Fig. 4B), consistent with the modest effect of siRNA-mediated NRF2 knockdown on cell growth after 3 days in culture (Fig. 3C). However, doxycycline-induced NRF2 downregulation potentiated the cytotoxic effect of lapatinib with almost no cells surviving after 12 days incubation, thereby providing evidence that NRF2 was essential for the acquisition of transient persister status (Fig. 4A, B). To prove that NRF2 was sufficient for mediating OAC cell survival in response to HER2 blocking agents, we tested the effect of increased NRF2 expression in OE19-PT cells by inducing KEAP1 knockdown by siRNA (Fig. 4C). OE19-PT cells transfected with siKEAP1 exhibited significantly less sensitivity to lapatinib than siControl transfected cells (Fig. 4D). Moreover, stable over-expression of full-length NRF2 or constitutively active NRF2 (caNRF2) – which lacks the N-terminal domain that binds its negative regulator KEAP1 [36] - restored the ability of OE19-PT cells to proliferate in the presence of lapatinib, as indicated by increased cell density (Fig. 4E, F, and Supplementary Fig. 5C, D).

**Fig. 5:**
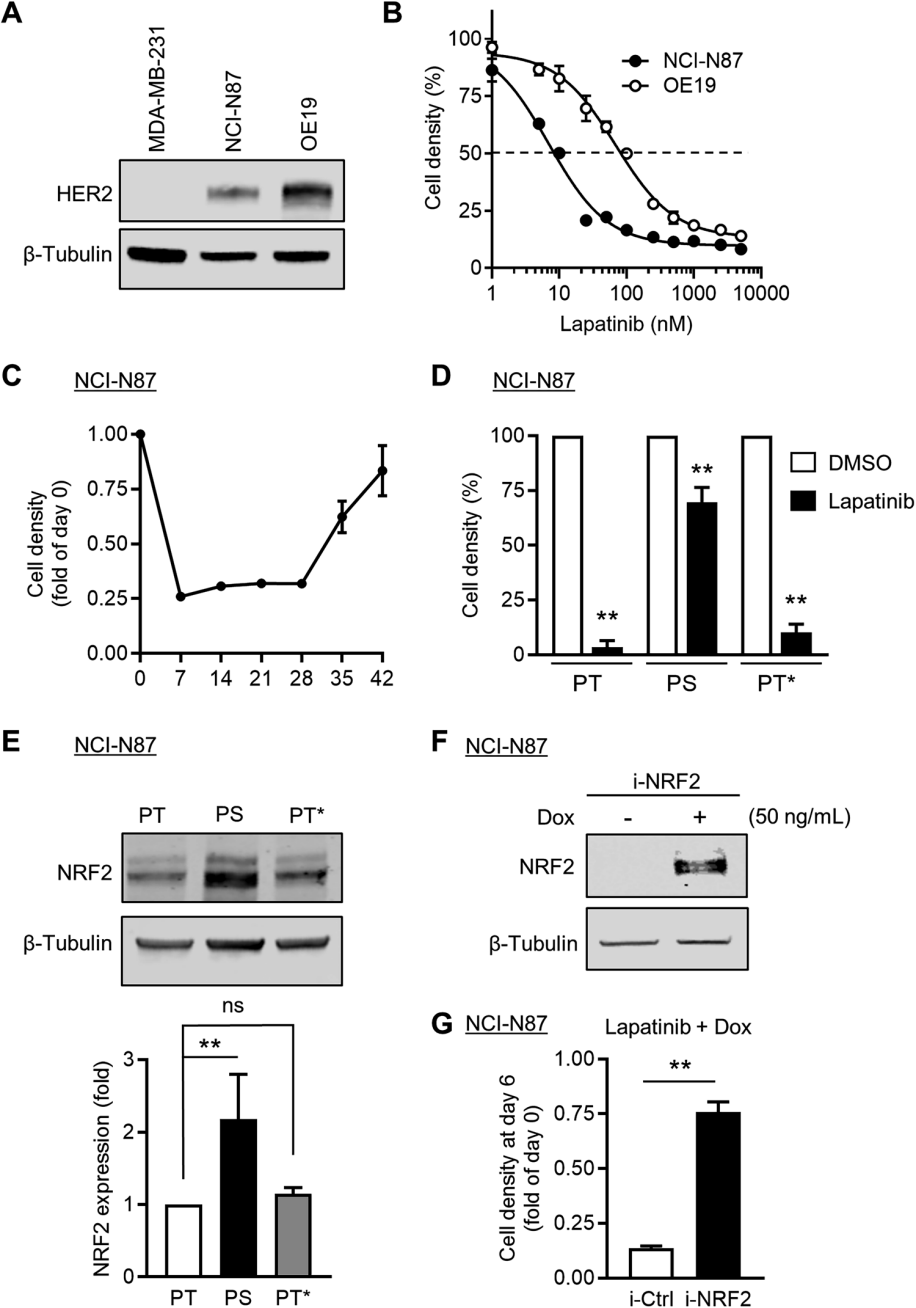
Continuous lapatinib treatment gives rise to NCI-N87 persistent cells. **A** Immunoblot analysis showing the level of HER2 expression in breast and oesophageal adenocarcinoma cell lines. MDA-MB-231 cells are triple negative breast cancer cells that lack HER2 expression. β-Tubulin expression was used as loading control. Similar results were obtained in two independent experiments. **B** Oesophageal adenocarcinoma cell lines were treated with various concentrations of lapatinib for 3 days as indicated, followed by crystal violet staining and quantification (*n* = 3). **C** NCI-N87 cells were treated with lapatinib (50 nM) for 42 days. Cell density was measured by crystal violet staining. The data are expressed as fold relative to the day lapatinib was added (day 0) (*n* = 3). **D** NCI-N87 parental (PT) cells were treated with lapatinib (50 nM) for 21 days and released from therapy for 1 day (NCI-N87-PS) or 14 days (OE19-PT*) prior to being mock treated with DMSO or exposed to lapatinib (50 nM) for 3 days. Cell density was measured by crystal violet staining (*n* = 3). **E** The level of NRF2 expression in NCI-N87 cell lines was quantified by immunoblot analysis (*n* = 4). β-Tubulin expression was used as loading control. **F** Immunoblot analysis showing increased level of NRF2 expression in NCI-N87 cells stably transduced with doxycycline inducible NRF2 (i-NRF2) encoding lentiviral particles after incubation with 50 ng/mL doxycycline (Dox) for 24 h. β-Tubulin expression was used as loading control. Similar results were obtained in two independent experiments. **G** NCI-N87 cells expressing an empty lentiviral construct (i-Ctrl) or a construct encoding inducible NRF2 (i-NRF2) were treated with lapatinib (50 nM) in the presence of doxycycline (Dox; 50 ng/mL) for 6 days. Cell density was quantified by crystal violet staining. The data are expressed as fold relative to the day lapatinib was added (day 0) (*n* = 3). Statistical analyses were performed by one-way ANOVA (**E**) or unpaired t-test (**D**, **G**).

We extended our observations to the gastro-oesophageal junction (GOJ) adenocarcinoma cell line, NCI-N87. Like OE19 cells, these cells express an elevated level of HER2 and are highly sensitive to lapatinib treatment (Fig. 5A, B). Moreover, we confirmed that NCI-N87 cells could enter into a DTP state. This was evident after 28 days of continuous lapatinib treatment, after which point the cells resumed proliferation (Fig. 5C). Consistent with their classification as DTPs, NCI-N87-PS cells that survived chronic exposure to lapatinib yielded HER2 inhibitor-sensitive progeny upon drug withdrawal for 2 weeks (Fig. 5D). We further demonstrated that the protein level of NRF2 was markedly increased in NCI-N87-PS cells, while N87-PT* cells which had re-acquired normal sensitivity to lapatinib treatment, exhibited a level of NRF2 similar to the parental cell line (Fig. 5E). We stably transduced NCI-N87 cells with a doxycycline-inducible NRF2 (i-NRF2) expressing construct. We found that doxycycline-induced NRF2 overexpression protected the cells against lapatinib cytotoxicity and restored cell proliferation, indicating the broader role of NRF2 in protecting gastro-oesophageal cancer cells (Fig. 5F, G).

Given that persistent cancer cells constitute a major cause of cancer treatment failure, these data collectively indicated that functional inhibition of the NRF2 pathway had the potential to be a novel strategy for OAC and possibly GOJ adenocarcinoma patients that have become resistant to HER2-targeted therapies.

### Targeting the NRF2 pathway achieves therapeutic efficacy in lapatinib-resistant OAC tumours

**Fig. 6:**
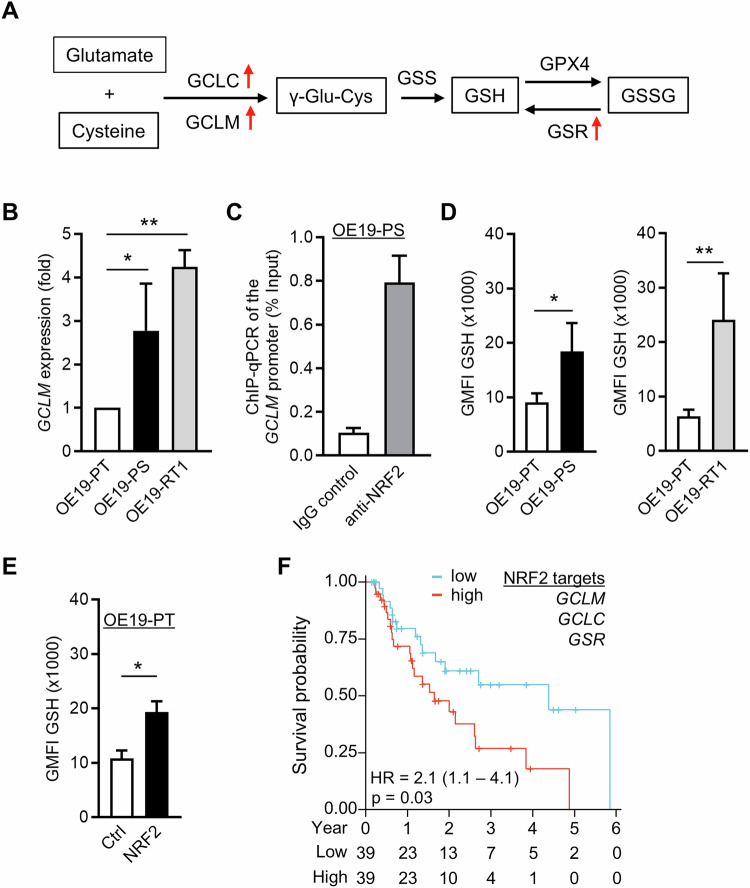
OE19-PS and OE19-R cells display increased levels of reduced glutathione. **A** Schematic representation of critical enzymes involved in glutathione metabolism. Red arrows indicate the genes encoding the enzymes upregulated in OE19-PS (treated with lapatinib for 35 days). **B** Detection of *GCLM* transcript levels in OE19-PT, OE19-PS and OE19-RT1 cells by qPCR (*n* = 3). **C** ChIP-qPCR shows endogenous binding of NRF2 to the *GCLM* promoter region (−43 to −203) in OE19-PS cells. Binding following immunoprecipitation with immunoglobulin (Ig) G control is also shown. (*n* = 2). **D**, **E** The amount of reduced glutathione (GSH) was measured by flow cytometry (*n* = 4). **F** Kaplan–Meier plot survival curves comparing subjects in the TCGA OAC cohort based on risk scores established from three NRF2 core target genes. Samples were divided into two groups, high (red) and low (blue), based on the median value of risk scores. Cox regression was used to calculate the hazard ratio (HR) and the *p*-value. Statistical analyses were performed by one-way ANOVA (**B**) or unpaired t-test (**D**, **E**).

Previous work had shown that NRF2 serves as a master transcriptional regulator of the cellular antioxidant response programme in cancer [28]. Strikingly, lapatinib treatment significantly increased the cellular level of reactive oxygen species (ROS) in OE19-PT cells after 24 h (Supplementary Fig. 6A). This was alleviated by incubation with the ROS scavenger *N*-acetyl cysteine (NAC) (Supplementary Fig. 6A). Moreover, NAC restored the ability of the cells to proliferate in the presence of lapatinib from day 2 onwards, in a concentration dependent manner (Supplementary Fig. 6B), suggesting that increased oxidative stress contributed to OE19-PT cell death following acute HER2 inhibition. We therefore speculated that NRF2 activation enabled OAC cells to survive persistent therapy and eventually develop resistance through its involvement in redox homeostasis. To test this hypothesis, we searched our curated clusters 2 and 9 for changes in gene expression linked to antioxidant pathways (Supplementary Fig. 2B and Supplementary Table 1). We found three NRF2 target genes encoding proteins involved in GSH synthesis and regeneration [37] whose expression reached a maximum after 35 days lapatinib treatment. These were the glutamate-cysteine ligase (GCL) catalytic (GCLC) and modifier (GCLM) subunits, and the glutathione reductase (GSR) (Fig. 6A).

We confirmed by qPCR that the level of the *GCLM* transcript was significantly higher in persistent and resistant OE19 cells compared with OE19-PT cells (Fig. 6B). Additionally, we demonstrated binding of NRF2 to the *GCLM* promoter region in OE19-PS cells by ChIP followed by qPCR (Fig. 6C). Consistent with these findings, persistent and resistant OE19 cells displayed a significantly higher level of reduced glutathione (GSH) compared with OE19-PT cells (Fig. 6D). Furthermore, stable overexpression of NRF2 in OE19-PT cells increased GSH levels provided strong direct evidence that NRF2 controlled glutathione metabolism in OAC cells (Fig. 6E). These results supported the idea that GSH was a crucial intracellular antioxidant for maintaining the survival of OAC cells adapted to persist HER2-targeted therapy. To assess the clinical relevance of the NRF2-mediated glutathione metabolic shift in OAC primary tumours, we used *GCLM*, *GCLC*, *GSR* transcript levels to calculate the risk score based on datasets from The Cancer Genome Atlas (TCGA). We observed that high risk score was correlated with worse survival of OAC patients (hazard ratio = 2.1; *p* value = 0.03) (Fig. 6F).

**Fig. 7:**
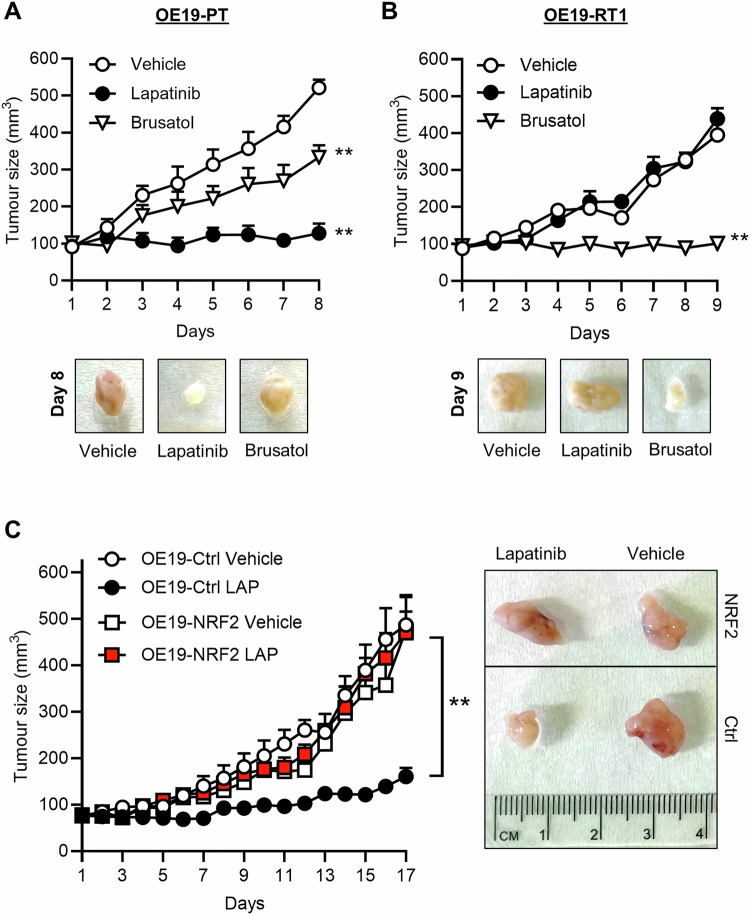
Resistance to lapatinib increases the sensitivity of OAC tumours to NRF2 inhibition by brusatol. OE19-PT (**A**), OE19-RT1 (**B**), and OE19-PT cells stably transduced with Ctrl (empty vector) or NRF2 encoding viral particles (**C**) were subcutaneously implanted into the flank of SCID mice. Animals carrying small tumours (approximately 80–120 mm^3^) subsequently received lapatinib (100 mg/Kg) or brusatol (2 mg/Kg) by oral gavage. Tumour size was measured daily for the duration of the experiment. The data are presented as the volume of tumour size (*n* = 4 or 5 mice in each group). Representative pictures of tumour grafts excised from mice sacrificed at the end of the experiment are shown. One-way ANOVA (**A**, **B**) and Wilcoxon rank sum test (**C**) were utilised to analyse statistical differences between groups.

To establish the significance of the NRF2 pathway as an actionable pathway to target OAC that have developed resistance to anti-HER2 therapies, we exploited an in vivo transplantation model of OAC where OE19-PT or OE19-RT1 cells were subcutaneously transplanted into 8 ~ 12-week female SCID recipient mice. We employed brusatol, a plant extract derived from *Brucea javanica*, to deplete NRF2 in OE19 cells through inhibiting protein translation (Supplementary Fig. 6C) [38]. Once tumours were detectable, randomised cohorts of mice were continuously treated with lapatinib or brusatol for the duration of the experiment. Lapatinib was utilised as a control to confirm the distinct sensitivity of OE19-PT and OE19-RT1 tumour xenografts to HER2-targeted therapy (Fig. 7A, B). We found that brusatol treatment completely halted the growth of OE19-RT1 tumours (Fig. 7B) while having a more moderate anti-tumour effect on OE19-PT grafts (Fig. 7A), as we would have predicted from our in vitro analysis of NRF2 knockdown in OE19 cell lines (Fig. 3C). In parallel, we transplanted NRF2-overexpressing and control OE19P-PT cells. Consistent with our in vitro data (Fig. 4E, F), NRF2 overexpression restored the normal growth of OE19-PT tumour grafts in the presence of lapatinib (Fig. 7C). Collectively, these observations provide strong evidence that the oxidative stress defence programme that emerges as a consequence of NRF2 pathway activation is crucial for resistant OAC cells to survive HER2-targeted therapy.

## Discussion

We previously reported that the therapeutic pressure exerted by continuous lapatinib treatment caused a global rewiring of chromatin accessibility and transcriptional regulation through HNF4A and PPARGC1A, as OE19 cells transitioned to a drug resistant state [17]. In this study, we propose that OE19 cells adapt and overcome the oxidative stress response to anti-HER2 targeted therapy by activating the antioxidant transcription factor NRF2 independently of KEAP1 degradation. Mechanistically, activated NRF2 re-established redox homeostasis after HER2 inhibition by enhancing de novo GSH biosynthesis and regeneration through transcriptionally upregulating the catalytic (GCLC) and modifier (GCLM) subunits of glutamate-cysteine ligase (GCL), as well as glutathione reductase (GSR) (Fig. 6). Importantly, our analysis based on TCGA patient data indicated that increased glutathione metabolism positively correlated with poor prognosis of OAC patients, which demonstrated the clinical relevance of our discovery (Fig. 6F). This finding is supported by a recent study which showed that neoplastic cells in Barrett’s oesophagus, the main precancerous condition of OAC, appear to overcome redox perturbations by constitutively overexpressing NRF2 [39]. Collectively, our results emphasise that the generation of persistent cells relies on a powerful anti-oxidant system to escape oncotherapy-induced cell death caused by accumulation of ROS [25].

In contrast to our findings, other studies have shown that drug tolerant persistent cells displayed increased vulnerability to ferroptosis induced by GPX4 inhibition, as a consequence of depleted GSH levels. In particular, lapatinib-induced HER2-expressing breast cancer persistent cells exhibited a broad downregulation of NRF2 target genes, including *GCLM* and *GSR*, compared with the parental cell line [21]. Likewise, glutathione metabolism was significantly suppressed in imatinib-derived gastrointestinal stromal tumour (GIST) persistent cells [22]. The reduced glutathione level in GIST persisters correlated with decreased GSR activity. Interestingly, Oren et al recently demonstrated that rare populations of persistent cells across multiple cancer types could re-enter the cell cycle under constitutive drug treatment for 10–14 days [18]. Compared with quiescent persisters, these cycling cells displayed higher expression of glutathione metabolism and NRF2 signatures, with lesser sensitivity to GPX4 inhibition by RSL3 [18]. However, OE19 persistent cells which begin to proliferate after 5 weeks of lapatinib treatment, remained exquisitely sensitive to RSL3 despite exhibiting a high level of NRF2 and GSH. These discrepancies may be attributed to variations in the abundance of polyunsaturated fatty acids (PUFAs)-containing phospholipids, which are a key factor influencing GPX4 dependency in the complex regulation of ferroptosis [40]. Notably, mesenchymal phenotypes that predispose cancer cells to ferroptotic death feature high levels of PUFAs [41, 42]. In light of this knowledge, it is interesting that p63 encoded by the *TP63* gene was one of the 5 most common transcription factors predicted to be involved in controlling upregulated DEG in OE19-PS cells (Fig. 1C). p63 functions as a key regulator of a quasi-mesenchymal cancer stem cell state known to be highly metastatic [43, 44]. Therefore, through p63-mediated transcriptional programming, it is possible that OAC DTPs exhibit a mesenchymal-like cell state highly sensitive to ferroptosis.

Additionally, the NRF2 pathway can rewire cancer cell metabolism in response to therapy-induced oxidative stress in other ways [45]. For example, persistent OE19 cells displayed a high level of the thioredoxin reductase 1 (*TXNRD1*) (Supplementary Fig. 2B and Supplementary Table 1), a key redox mediator essential to re-activate dormant breast tumour cells and promote recurrent tumour formation [46]. Also upregulated in persistent OE19 cells was Niemann-Pick C1-like 1 (*NPC1L1*), which has previously been implicated in the adaptation of multidrug resistant cancer cells entering a drug-tolerant persistent state by promoting uptake of vitamin E [47], (Supplementary Table 1). In another study, NRF2-induced expression of the transient receptor potential cation channel subfamily A (*TRPA1*), a neuronal redox-sensing channel that upregulates a Ca^2+^-dependent anti-apoptotic pathway, promoted oxidative stress tolerance of human breast and lung cancer cells during tumourigenesis and in response to chemotherapies [48]. In future, further functional analyses of the large transcriptional network of antioxidant enzymes downstream of NRF2 will be necessary to reveal the broad metabolic changes responsible for mediating the survival of oesophageal cancer cells after HER2 downregulation.

From a clinical perspective, the concept of persistent cancer cells relates to a non-genetic adaptive mechanism that allows tumours to evade therapy and disease recurrence [19]. Therefore, our findings that NRF2 activation in OAC occurs independently of genomic alterations in the KEAP1-NFR2 axis provides an attractive therapeutic option for this disease. In agreement with the benefit of targeting NRF2 to eliminate the oesophageal cancer cell population that persists after anti-HER2 therapies, we demonstrated that administration of brusatol blocked the growth of lapatinib resistant oesophageal tumour grafts (Fig. 7). The anti-tumour effect of brusatol has also been observed in tumour graft models of HER2-expressing breast and ovarian cancer through induced ROS generation and enhanced ferroptotic death [49]. Conversely, we found that brusatol therapy only partially impaired the growth of lapatinib sensitive OE19 tumour grafts, thereby minimising off-target effects of brusatol on global protein synthesis in resistant tumours [50]. Overall, these results highlight NRF2 as a promising target for treating OAC patients resistant to HER2 therapies. Consequently, there is an urgent need to develop more selective NRF2 inhibitors that do not interfere with the translational machinery to further validate the therapeutic potential of our discoveries in overcoming HER2 resistance in OAC patients.

## Materials and methods

### OE19 persister and resistant cell generation and treatments

Around 3 × 10^6^ parental (PT) OE19 cells were seeded in 10-cm plates and allowed to adhere overnight before being treated with 500 nM lapatinib-containing media for 35 days to generate drug-tolerant persister (PS) OE19 cells. Cells that had survived after 35 days were expanded in lapatinib media for 12 months to generate a population of resistant (RT1) cells. A clearly separated individual resistant (RT2) colony was isolated from OE19-RT1 cells seeded at low density. The medium with fresh lapatinib was replaced every 2–3 days. For follow-up explorations, the cells were replated after trypsinisation and allowed to adhere overnight in lapatinib-free culture medium. The medium was changed the following day to drug-containing media until the end of the experiment. The following drugs were used: 500 nM lapatinib (Cell Signalling Technology #12121), 10 nM neratinib (APExBIO #A8322), 10 µg/mL trastuzumab (Genentech), 500 nM RSL3 (Selleckchem #S8155), 1 µM Ferrostatin-1 (Selleckchem #S7243) and 10 nM brusatol (Selleckchem #S7956). Cell sensitivity to the treatments was performed in 24 well plates (5 × 104 OE19 cells and 2.5 × 105 NCI-N87 cells per well) by measuring optical density (OD) at 570 nm after fixation in methanol and staining with crystal violet (Sigma-Aldrich).

### siRNA transient transfection

Negative control siRNA (siScr #4390843) and siRNA targeting *KEAP1* (#s18981) were purchased from ThermoFisher Scientific. The siRNAs targeting *NRF2* (#L-003755-00-0005) and *GCLM* (#l-011670-01-0005) were purchased from Dharmacon as ON-TARGETplus SMART pools. All siRNAs were transfected with the Lipofectamine RNAiMAX reagent (ThermoFisher Scientific #13778150) in sub-confluent cells cultured in 24 well (5 pmol per well) or 6 well (25 pmol per well) plates, according to the manufacturer’s instructions.

### Lentivirus-mediated NRF2 silencing and NRF2 over-expression

To generate doxycycline-dependent inducible shRNAs, double-stranded oligonucleotides targeting the last exon shared by all spliced variants of *NRF2* transcripts (5’-AGCACCTTATATCTCGAAGTT-3’) and non-targeting control (5’-CCTAAGGTTAAGTC GCCCTCG-3’) were chemically synthesised by Integrated DNA Technologies and subcloned using AgeI and EcoRI restriction enzymes in the Tet-pLKO-puro lentiviral transfer plasmid (Addgene #21915). For doxycycline-dependent inducible expression, NRF2 cDNA was subcloned into the bicistronic lentiviral expression vector pCHD-TRE3GS-MCS-EF1a-puromycin (a gift from Stuart Cain, University of Manchester, in which target gene expression is driven by the TRE3GS promoter and puromycin expression is driven by the EF-1α core promoter) using EcoRI and NheI restriction enzymes. Constitutively activated (ca) NRF2 lacking amino acids 1-81 and full length NRF2 cDNAs were generated from the NRF2 template (Addgene #21555), and subcloned into the pLV-EF1a-IRES-Puro plasmid (Addgene #85132) using BamHI and EcoRI restriction enzymes. Sequences of oligonucleotides to make these constructs are available in Supplementary Table 2. Lentiviral particles were generated with HEK293T cells as previously described [51]. Infected cells were selected by incubation with 2 µg/mL puromycin until no live cells remained in the non-infected group (at least 3 days). Resistant colonies were pooled and expanded in puromycin-free containing medium.

### OAC tumour grafts

A total of 2 × 10^6^ cells in 100 μL of basement membrane matrix (Gibco #A1413201):PBS (1:1) were subcutaneously implanted into the rear flank of immunodeficient SCID female mice. Once tumours had reached a palpable size [around 80 mm^3^ by caliper measurements using the formula: 0.5 × (length × width × depth)], the mice were simply randomised into groups and treated with 100 μL of vehicle, 100 mg/Kg lapatinib (APExBIO #A8218) or 2 mg/Kg brusatol. Lapatinib and brusatol were dissolved in ultrapure water containing 1% Tween-80 and 5% hydroxypropyl methylcellulose just before being administered by oral gavage every other day. Tumour size was subsequently determined every day in a treatment-blinded manner. The demonstration of comparable tumour sizes in at least four animals per treatment group was considered definitive evidence of the effect, effectively ruling out artifacts related to biological variability. Mice were euthanised when obvious differences between each group were obtained.

### Bioinformatics analyses

Clinical OAC RNA-seq raw count and prognosis data were downloaded from the TCGA portal (https://portal.gdc.cancer.gov/). For time-course RNA-seq data [17], Fastp (version 0.20.0) was utilised for trimming adaptors and removing low-quality reads to yield clean reads. Alignment of these high-quality clean reads to the human reference genome (hg38) was performed using the STAR (version 2.7.9a). The FeatureCounts (version 2.0) facilitated the acquisition of raw gene-level mRNA read counts, forming the basis of the mRNA expression profile. Annotation of the mRNA was conducted using the Ensembl GTF gene annotation database (version 104). DESeq2 package was used to assess fold changes and p-values between the two groups [52].

GSVA tool was used to evaluate the NRF2 activation score based on the list of gene targets [53]. For A549 cells NRF2 ChIP-seq data [32], adaptor trimming and low-quality read removal were performed using Fastp software, resulting in high-quality clean reads. The clean reads were then aligned to the human hg38 reference genome using Bowtie2 software (v2.2.4). Peak calling was conducted using MACS2 software (v2.2.7.1). Peak annotation was performed with the ChIPSeeker R package (v1.30.3). Motif identification was carried out using Homer software (v4.11).

For ATAC-seq data [17], analyses were conducted in accordance with the Harvard FAS Informatics ATAC-seq Guidelines. Cleaned reads were aligned to the human reference genome (hg38) using Bowtie2 software. The ATACseqQC R package (v1.22.0) was employed to calculate and plot the fragment size distribution. Peak calling was performed using Genrich software (v1.30.3). The ChIPseeker R package (v1.30.3) was used for annotating the enriched peaks. Homer software was used to identify motifs. Differentially enriched region analysis between two sample groups was conducted using the MANorm2 R package. ARE motif (5’-TGACNNNGC-3’) was scanned and extracted using fimo with default parameters [54]. ChIP-seq identified NRF2 binding sites and ARE motif sites were mapped to ATAC-seq data to plot the heatmaps.

To develop the prognostic model, univariate Cox regression was used to identify survival-associated genes with *q*-value < 0.1. Candidate genes were further refined via random survival forest to prioritise top-ranked features based on minimal depth and variable importance. We employed filtered core NRF2 gene targets to calculate the risk score using the formula risk score = ∑(xi × βi), where xi represents the gene expression value and βi is the coefficient index, via a multivariate Cox regression model. Patients were categorised based on the median risk score, and differences in survival between the high-risk and low-risk groups were evaluated using Kaplan–Meier analysis. The survminer package was used for visualizing the results. All analysis were performed in R (version: 4.2.2).

### Statistical analyses

Data were plotted as averages of biological repeats and error bars indicate standard deviation (SD). The GraphPad Prism 10.0 software was employed for all statistical analyses using unpaired t-test for direct comparison of two conditions or one-way ANOVA for comparison of multiple conditions. Differences were considered statistically significant when the p-value was less than 0.05. *p ≤ 0.05; **p ≤ 0.01; ns non-significant.

## Supplementary information

Supplementary Information

Supplementary Figures 1 to 6

Supplementary Table 1

Supplementary Table 3

## Data Availability

Data (RNA and ATAC sequencing of OE19 cells) utilised in this study have been deposited in the EMBL’s European Bioinformatics Institute database (https://www.ebi.ac.uk/) with the accession numbers E-MTAB-10304 and E-MTAB-10314. Coding scripts are available upon request.
