# Supplementary material for: NRF2-mediated persistent adaptation of oesophageal adenocarcinoma cells to HER2 inhibition
Source: Oncogene. 2025 Jun 5;44(33):2929–41. doi: 10.1038/s41388-025-03459-0 (PMC12336050; doi:10.1038/s41388-025-03459-0)
Supplement: Supplementary file 1 — Supplementary Information [file 41388_2025_3459_MOESM1_ESM.docx]

**Supplementary Information for:**

**NRF2-mediated persistent adaptation of oesophageal adenocarcinoma cells to HER2 inhibition**

**Running title:** Targeting lapatinib-resistant oesophageal adenocarcinoma by inhibiting NRF2

Wei Zhang, Jiaqing Lang, Sorayut Chattrakarn, Chun Wai Wong, Shiyang Li, Karmern Kan, Hongcai Liu, Wenchao Gu, Jingwei Zhang, Jukka Westermarck, Alan J Whitmarsh, Andrew D Sharrocks^3^, and Cathy Tournier^1^*****

**Supplementary Material and Methods**

**Supplementary Tables 1 to 3**

**Supplementary Figures 1 to 6**

**Supplementary Materials and Methods**

**Cell lines and cell culture**

OE19 cells were purchased from the European Collection of Authenticated Cell Cultures (ECACC). NCI-N87, MDA-MB-231 and HEK293T cells were purchased from American Type Culture Collection (ATCC). OE19 and NCI-N87 cells were cultured in RPMI-1640 (Sigma-Aldrich #R8758), and MDA-MB-231 and HEK293T cells were cultured in DMEM (Sigma-Aldrich #D6429), each supplemented with 10% foetal bovine serum (FBS) (ThermoFisher Scientific #10500064). Cell authentication was not routinely conducted given that all cell lines were utilised for a maximum of 20 passages before thawing another aliquot of the same stock to maintain the original phenotype. Mycoplasma testing was routinely performed as described [1].

**Protein extraction and immunoblot analysis**

Cells cultured in six-well plates (2.5 x 10^5^ cells per well) were lysed in RIPA buffer (Sigma-Aldrich #R0278) supplemented with protease and phosphatase inhibitors (ThermoFisher Scientific #87786 and #78420). Protein concentrations were quantified by DC^TM^ protein assay (Bio-Rad #500-0112). Cellular fractionation was performed as previously described [2] Extracts were resolved by 4-20% precast polyacrylamide gels (Bio-Rad #4568093) and subjected to immunoblot analyses with the following antibodies: HER2 (CST #2165), NRF2 (ABclonal #A21176), KEAP1 (Proteintech #10503-2-AP), GSK-3β (ABclonal #A3174), P-GSK-3β Ser9, (ABclonal #AP1088), GCLM (Abcam #EPR6667), GAPDH (Abclonal #A19056), Histone H3 (Abcam #ab176842), and β-Tubulin (Abcam #ab6046). Immunocomplexes were detected by using the Odyssey® CLx Imaging system with IRDye® 800CW anti-Rabbit IgG secondary Antibody (LI-COR #926-32213). Fluorescent signals were quantified using Empiria studio v1.1. Housekeeping proteins (β-Tubulin or GAPDH) were employed to normalise protein loading between samples.

**RNA extraction and qPCR**

Total RNA was extracted by using an RNA Purified Total RNA Extraction Kit (Qiagen #74104), reversed transcribed to cDNA using RNA-to-cDNA kit (ThermoFisher Scientific #4387406) and quantified by qPCR using the Takyon^TM^ ROX Probe Master Mix from Eurogentec (#UF-RPMT-B0701). TaqMan probes for the following genes were purchased from ThermoFisher Scientific: *NFE2L2* (Hs00232352_m1), *GCLM* (Hs00978072_m1), *ABCC2* (Hs00166123_m1), *AKR1C1* (Hs04230636_sH), *G6PD* (Hs00166169_m1), *HMOX1* (Hs01110250_m1), *TXNRD1* (Hs00917067_m1) and *RPLP0*. Results were analysed using the 2^-ΔΔCT^ method. The level of expression of mRNA was normalised to *RPLP0*.

**ChIP-qPCR**

Roughly 4 × 10⁶ cells were cross-linked using 1% formaldehyde for 10 min at room temperature, followed by quenching with glycine, prior to being collected and lysed as previously described [3, 4]. Chromatin was fragmented by sonication to obtain fragments of 150-600 bp. Sonication efficiency was assessed by agarose gel electrophoresis. Dynabeads A (ThermoFisher #10002D) were pre-blocked with BSA and incubated overnight at 4°C with 5 μg of either NRF2 antibody (CST #12721) or IgG control (CST #2729). Chromatin fragments were incubated with antibody-bound beads overnight at 4°C. Immunoprecipitated DNA was purified using the QIAquick PCR Purification Kit (Qiagen #28104). Purified DNA was amplified by PCR with primers covering the *GCLM* promoter region (–43 to –203) (Supplementary Table 2) [5], and quantified using SYBR Green-based qPCR (Qiagen #204143). Total DNA served to measure the input.

**Flow cytometry**

Cells were cultured in 6-cm dishes (5 x 10^5^ cells per dish) and collected after treatment by trypsinisation. To assess dead versus live cells, the collected cells were washed with PBS and then processed by using the Zombie viability kit (BioLegend #423108) according to the manufacturer’s instruction. For distinguishing non-proliferative and proliferative cells by nuclear staining of Ki-67, cells were subsequently fixed and permeabilised (fixation and permeabilisation solution from Biosciences #554722), and incubated overnight with AlexaFluor700-conjugated Ki-67 antibody (eBioscience #SolA15) diluted in permeabilisation buffer (eBioscience #00-8333-56). For GSH measurements, cells were incubated with 100 µM monochlorobimane (MCB, Sigma #69899) for 30 min before being collected by trypsinisation. Fluorescence was acquired on a BD Fortessa flow cytometer and analysed using FlowJo software v10 (Tree Star).

**Reactive oxygen species (ROS) detection by immunofluorescence**

Cells cultured in 6-well plates (2.5 x 10^5^ cells per well) were incubated with the ROS Deep Red dye (Abcam #ab186029) and visualized with the EVOS cell imaging system (ThermoFisher Scientific).

1 - Young L, Sung J, Stacey G, Masters JR. Detection of Mycoplasma in cell cultures. Nat Protoc. 2010;5:929-34.

2 - Suzuki K, Bose P, Leong-Quong RY, Fujita DJ, Riabowol K. REAP: A two minute cell fractionation method. BMC Res Notes. 2010;3:294.

3 - Geisberg JV, Struhl K. Quantitative sequential chromatin immunoprecipitation, a method for analyzing co-occupancy of proteins at genomic regions in vivo. Nucleic Acids Res. 2004;32:e151.

4 - Ji Z, Donaldson IJ, Liu J, Hayes A, Zeef LA, Sharrocks AD. The forkhead transcription factor FOXK2 promotes AP-1-mediated transcriptional regulation. Mol Cell Biol. 2012;32:385-398.

5 - Gong S, Zhang A, Yao M, Xin W, Guan X, Qin S, Liu Y, Xiong J, Yang K, Xiong L, He T, Huang Y, Zhao J. REST contributes to AKI-to-CKD transition through inducing ferroptosis in renal tubular epithelial cells. JCI Insight. 2023;8:e166001.

**Supplementary Table 1**

Lists of curated NRF2 gene targets from human NSCLC (108 genes) and lymphoblastoid cells (207 genes), and overlapping target genes (67 genes) between clusters 2 and 9 (C2+C9), NSCLC and lymphoblastoid signatures (10 genes, written in purple), C2+C9 and NSCLC signature (30 genes, written in green), C2+C9 and lymphoblastoid signature (27 genes, written in grey).

**Supplementary Table 2**

**Oligonucleotides used in this study**

|  | **for constructing Tet-pLKO-Puromycin-shNRF2** |
| --- | --- |
| Forward | 5’ - CCGGAGCACCTTATATCTCGAAGTTCTCGAGAACTTCGAGATAT  AAGGTGCTTTTTTG - 3’ |
| Reverse | 5’ - AATTCAAAAAAGCACCTTATATCTCGAAGTTCTCGAGAACTTCG  AGATATAAGGTGCT - 3’ |
|  | **for constructing Tet-pLKO-Puromycin-shControl** |
| Forward | 5’ - CCGG**CCT**AAGGTTAAGTCGCCCTCGCTCGAGCGAGGGCGACTTA  ACCTTAGGTTTTTG - 3’ |
| Reverse | 5’ - AATTCAAAAACCTAAGGTTAAGTCGCCCTCGCTCGAGCGAGGG  CGACTTAACCTTAGG - 3’ |
|  | **for constructing pCHD-TRE3GS-NRF2-EF1a-Puromycin** |
| Forward | 5’ - ACCCTCGTAAATTAATTAAGCTAGCATGATGGACTTGGAGCTG - 3’ |
| Reverse | 5’ - GGAGCGATCGCAGATCCTTTTAGAATTCCTAGTTTTTCTTAACAT  CTGGCTTC - 3’ |
|  | **for constructing pLV-EF1a-IRES-Puromycin-NRF2** |
| Forward | 5' - TCCATTTCAGGTGTCGTGAGGATCCATGATGGACTTGGAGCTGCC  GCCGC - 3’ |
| Reverse | 5’- TAGAGCGGCCGCCCTCGAGGAATTCCTAGTTTTTCTTAACATCTG  GCTTC - 3’ |
|  | **for constructing pLV-EF1a-IRES-Puromycin-caNRF2** |
| Forward | 5’- TCCATTTCAGGTGTCGTGAGGATCCATGCAGCACATCCAGTCAGA  AACCAGTG - 3’ |
| Reverse | 5’- TAGAGCGGCCGCCCTCGAGGAATTCCTAGTTTTTCTTAACATCTGG  CTTC - 3’ |
|  | **for amplifying the *GCLM* promoter region (–43 to –203) by PCR** |
| Forward | 5’ – GCCACGCTCTCTCGACC - 3' |
| Reverse | 5'-AGCCGAGAAAGTGCTTCGTA-3' |

**Supplementary Table 3**

Lists of manually curated pro-ferroptotic and anti-ferroptotic markers from the FerrDb database.

**Supplementary Figure legends**

**Supplementary Fig. 1: The development of persistence correlates with NRF2 activation.**

**A** OE19 cells were cultured in complete media with 500 nM lapatinib and harvested at day (d) 1, 7 and 35 for RNA sequencing (n = 3) [17]. Samples at day 0 correspond to cells treated with DMSO for 24 h. DEGs (vs. d0, p < 0.01) were selected for mfuzz time-course analysis. Genes were grouped into 9 clusters, according to their expression pattern over the time course of lapatinib treatment. **B** OE19 cells were treated with tBHQ (50 μM) for 6 h, followed by qPCR detection of NRF2 target genes. One-way ANOVA was utilised to analyse statistical differences between tBHQ versus mock treated cells with DMSO (n = 3). **C** Genetic perturbation similarity analysis (GPSA) plot. DEGs (d35 vs. d1) were compared to a curated database of 3048 RNA-seq datasets from 1458 gene knock downs in human cell lines. The genetic perturbation similarity index (GPSI) scores of the five highest ranked genetic perturbation causing the most similar transcriptional changes to that detected in OE19 cells treated with lapatinib for 35 days are shown.

**Supplementary Fig. 2. Expression of NRF2 target genes are increased with the acquisition of lapatinib tolerance.**

**A** Venn-diagram showing the numbers of overlapping genes between NRF2 gene signatures from NSCLC and lymphoblastoid cells [30, 31] and lapatinib-induced gene expression clusters 2 and 9 (C2+C9; see Supplementary Fig. 1A). **B** Heatmap representing the transcriptional expression profile of the indicated NRF2-regulated genes over the time course of lapatinib (500 nM) treatment of OE19-PT cells. The NRF2-regulated genes were identified from the intersections between NRF2 signatures and DEGs in clusters 2 and 9; warm colours (shade of red) signify high expression and cool colours (shades of blue) low expression based on row z-scores. Each column represents a sample (n = 3). **C** RNA sequencing datasets were utilised to assess the level of ferroptotic activity in OE19-PT and OE19-PS cells by GSVA of pro-ferroptotic and anti-ferroptotic gene sets from the FerrDb database (Supplementary Table 3). **D** Detection of *GPX4* transcript levels in OE19-PT and OE19-PS by RNAseq (n = 3). Unpaired t-test was utilised to analyse statistical differences (C, D).

**Supplementary Fig. 3: Generation of lapatinib-resistant OE19 cells.**

**A** OE19-PT, OE19-RT1 and OE19-RT2 cell lines were treated with various concentrations of lapatinib for 3 days as indicated, followed by crystal violet staining and quantification (n = 3). IC50 estimates are indicated in the table. **B** OE19-PT, OE19-RT1 and OE19-RT2 cell lines were treated with lapatinib (500 nM), neratinib (10 nM), or trastuzumab (10 μg/mL) for 3 days or incubated with RSL3 (500 nM) for 24 hours. Cell density was measured by crystal violet staining (n = 3). The same data for OE19-PT cells are presented in Fig. 1A and only utilised in this panel for reference. One-way ANOVA was utilised to analyse statistical differences between treated versus mock treated cells with DMSO.

**Supplementary Fig. 4: Quantification of the immunoblots presented in Fig. 3A.**

Changes in protein expression relative to that of the housekeeping protein GAPDH are presented in fold (n = 3). Statistical analyses were performed by one-way ANOVA (PS, PT* vs. PT) or unpaired t-test (RT vs. PT).

**Supplementary Fig. 5: Analysis of NRF2 expression in OE19 cell lines.**

**A** OE19-PT cells were incubated with the GSK3 inhibitors Tideglusib (Tid, 5 μM) or Laduviglusib (Lad, 5 µM and 10 µM) for 24 h. Protein lysates were analysed by immunoblot with the indicated antibodies. GAPDH expression was used as loading control. Similar results were obtained in two independent experiments. **B** *NRF2* transcript level in OE19-PT cells after silencing by siRNA for 24 h was measured by qPCR (n = 3). The reduction in NRF2 expression in OE19-PS and OE19-RT cell lines transduced by siNRF2 is shown by immunoblot analysis. Mock silenced (siScr treated) cells were employed as negative controls. **C** Immunoblot analysis showing increased level of caNRF2 expression in OE19-PT cells stably transduced with caNRF2 encoding lentiviral particles. *Indicates degradation products of caNRF2. β-Tubulin expression was used as a loading control. Similar results were obtained in three independent experiments. **D** Quantification of cell density by crystal violet staining of Ctrl (empty vector) and caNRF2 expressing OE19-PT cells treated with lapatinib (500 nM) for 6 days. The data are expressed as fold relative to the day lapatinib was added (day 0) (n = 3). Unpaired t-test was utilised to analyse statistical differences (B, D).

**Supplementary Fig. 6: Lapatinib treatment induces oxidative stress in OE19 cells.**

**A** OE19-PT cells were mock treated with DMSO or treated with lapatinib (LAP; 500 nM) for 24 hours in the presence or absence of NAC (1 mM). ROS were detected by live cell imaging after cell incubation with the ROS Deep Red dye. **B** OE19-PT cells were treated with lapatinib (LAP; 500 nM), alone or in combination with increasing concentrations of NAC. Cell density was quantified by crystal violet staining. The data are expressed as fold of mock treated cells plated at day 0 (n = 3). Statistical analyses were performed at day 8 using one-way ANOVA. **C** Immunoblot analysis of NRF2 expression in OE19 cell lines treated with brusatol (10 nM) for 5 h. β-Tubulin expression was used as loading control. Similar results were obtained in three independent experiments.
