# Supplementary material for: NRF2-mediated persistent adaptation of oesophageal adenocarcinoma cells to HER2 inhibition
Source: Oncogene. 2025 Jun 5;44(33):2929–41. doi: 10.1038/s41388-025-03459-0 (PMC12336050; doi:10.1038/s41388-025-03459-0)
Supplement: Supplementary file 2 — Supplementary Figures 1 to 6 [file 41388_2025_3459_MOESM2_ESM.pdf]

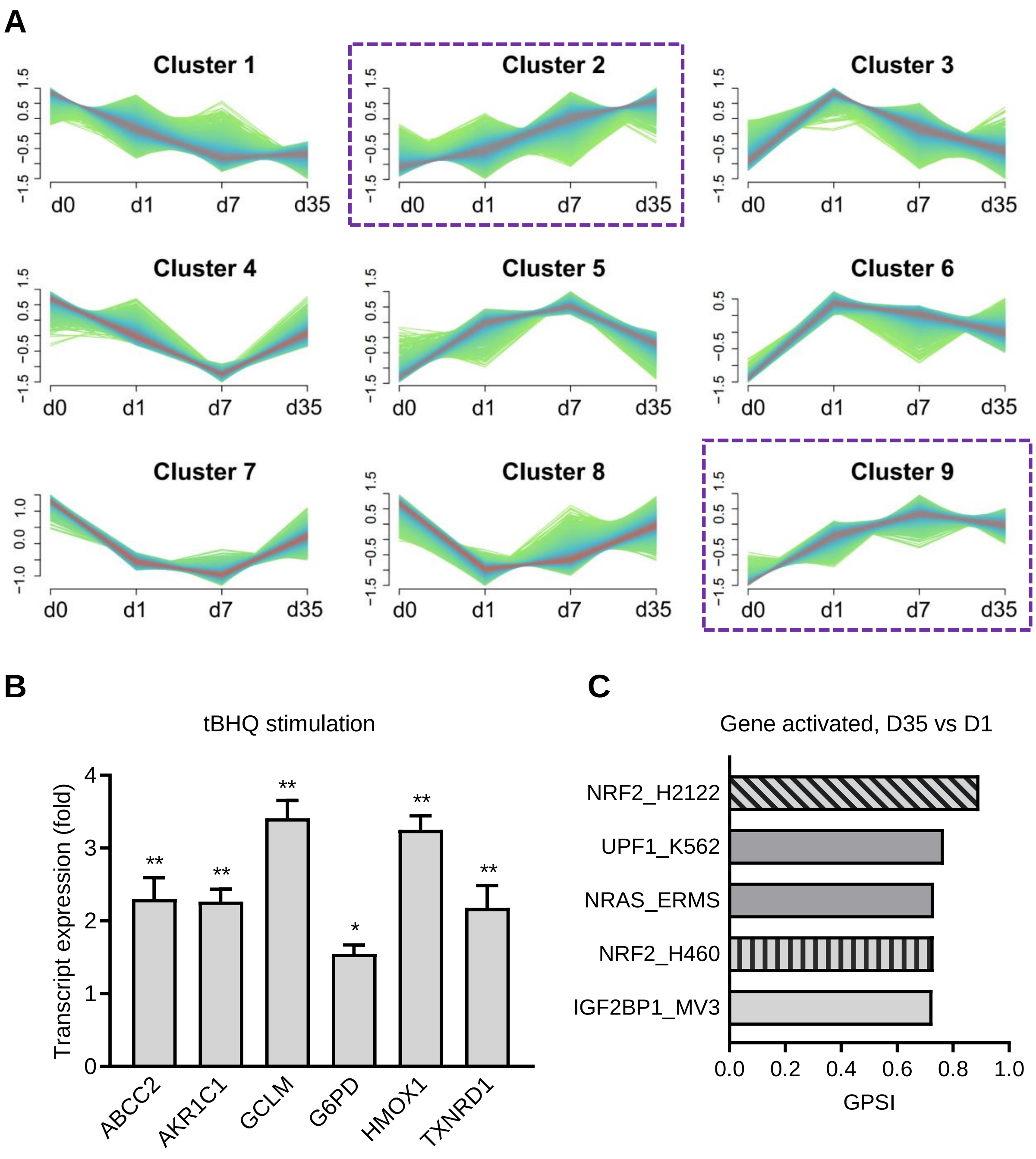

Supplementary Figure 1

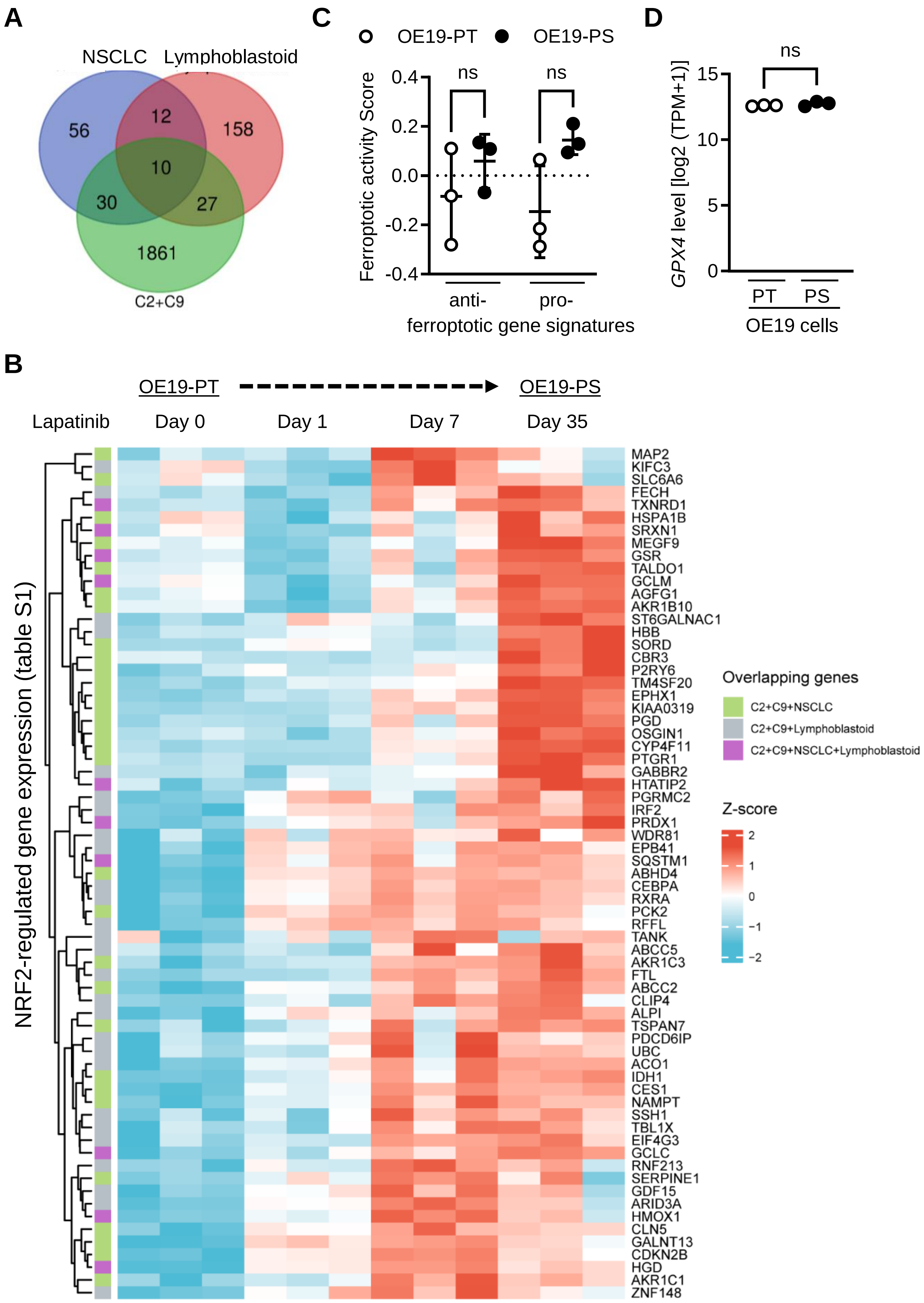

Supplementary Figure 2

A

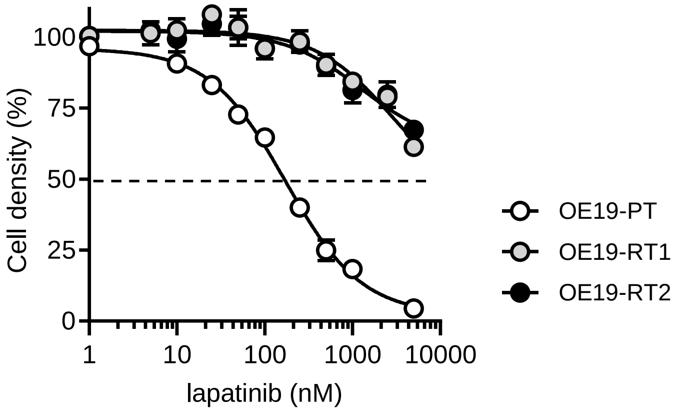

Type	IC50
OE19-PT	100 nM
OE19-RT1	>1 µM
OE19-RT2	>1 µM

B

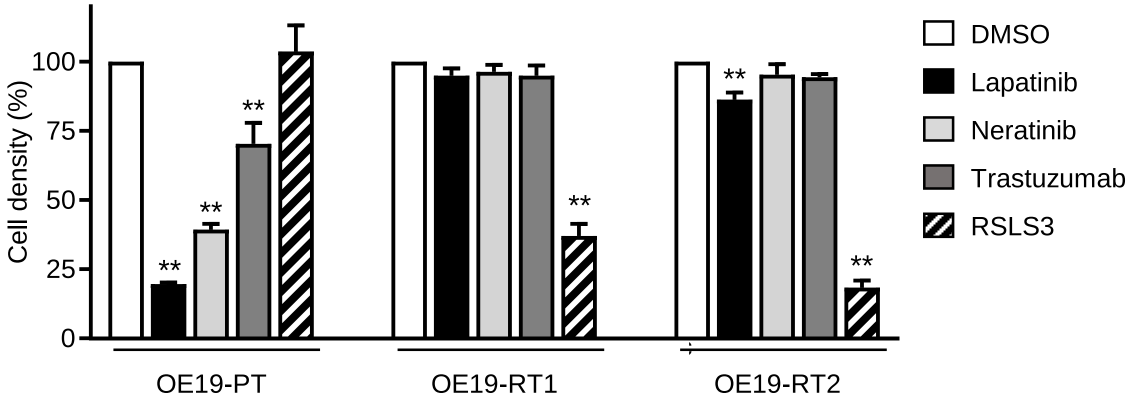

Supplementary Figure 3

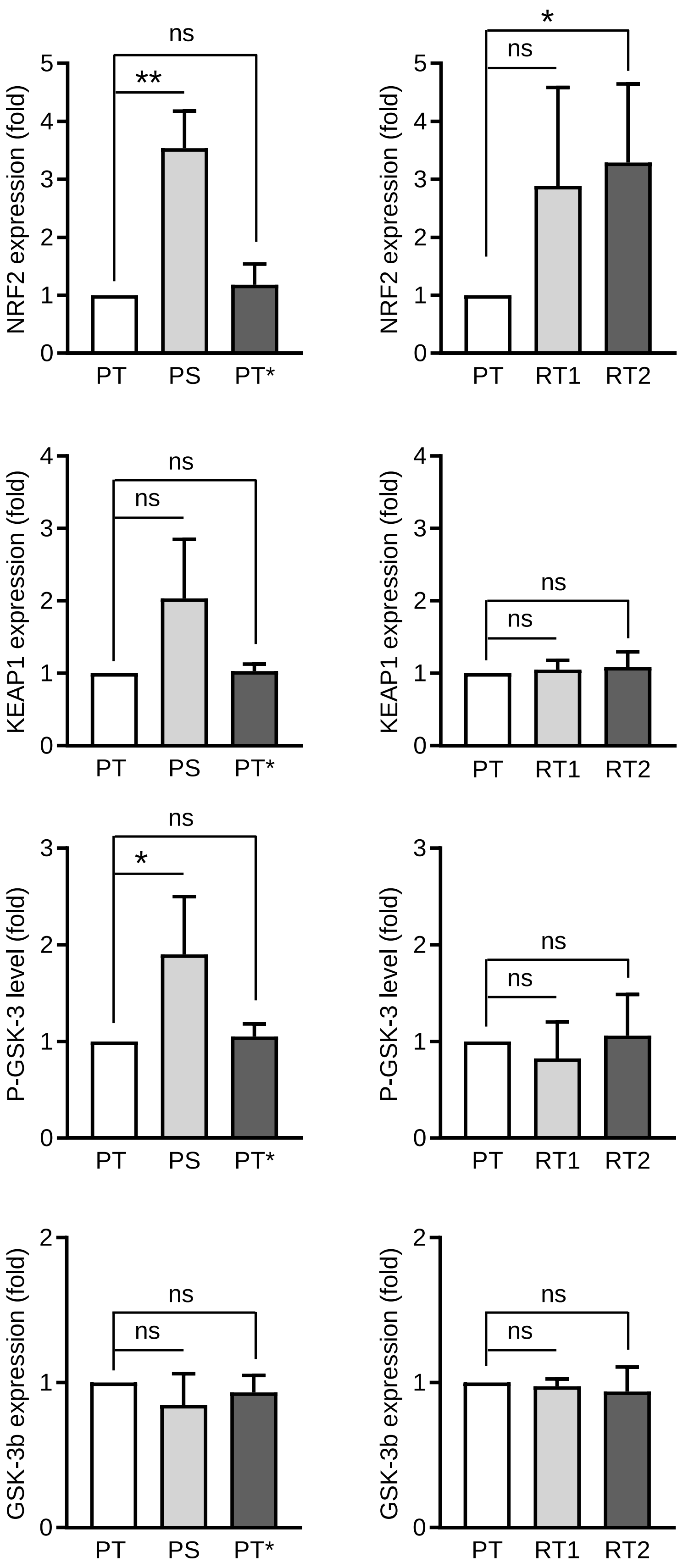

Supplementary Figure 4

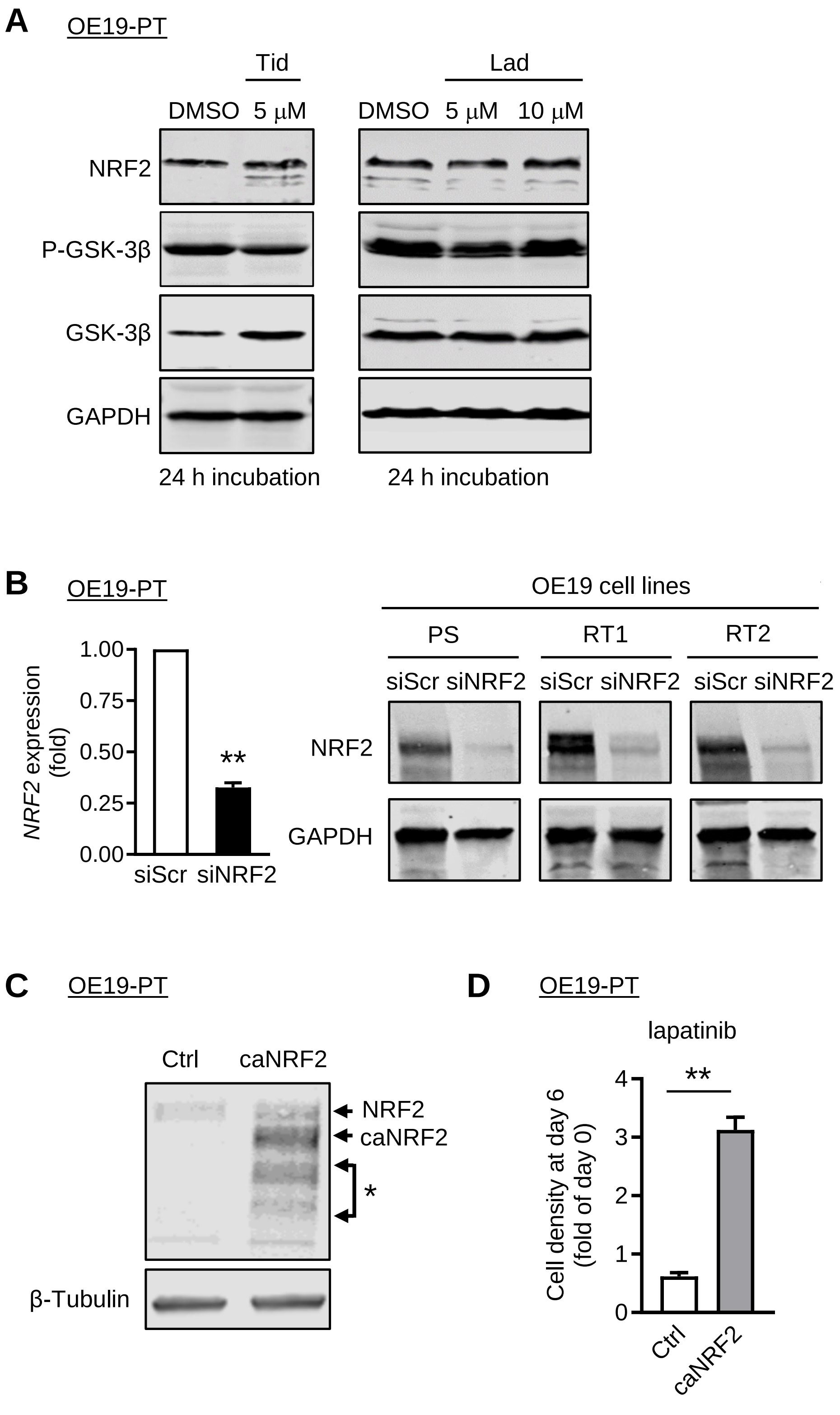

Supplementary Figure 5

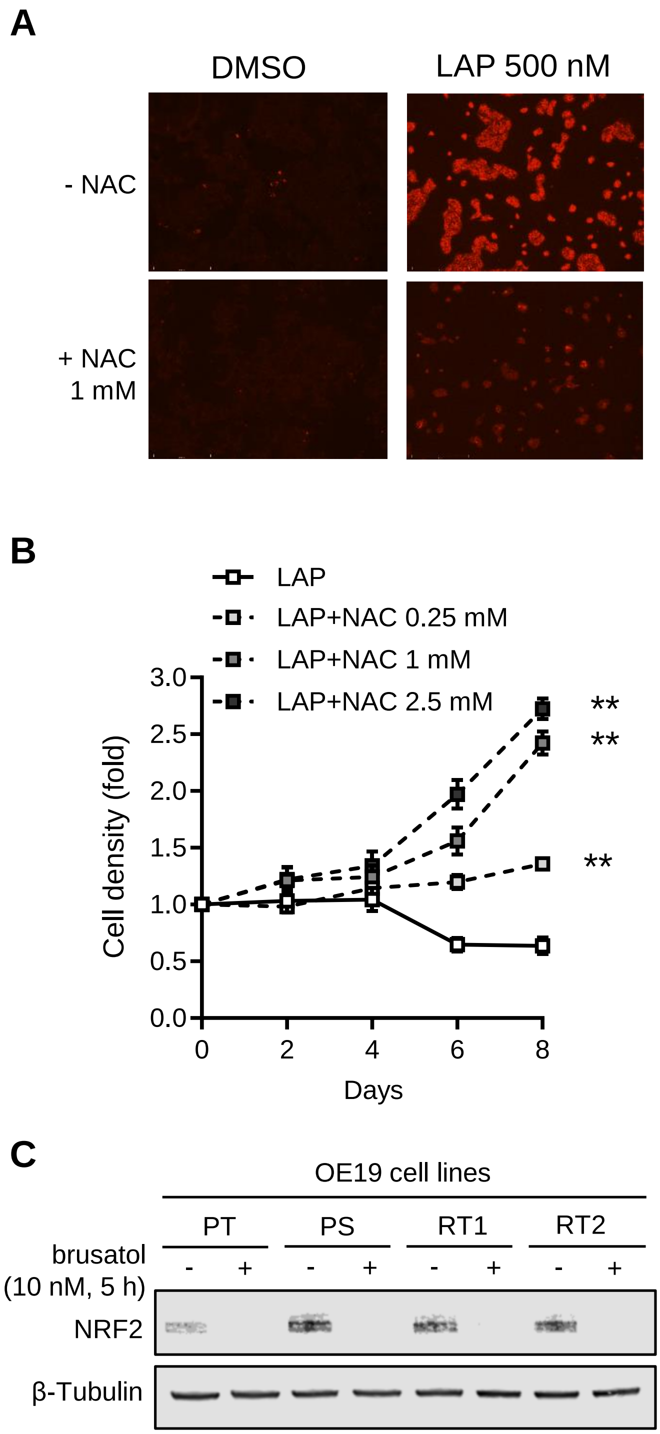

Supplementary Figure 6
